# Seasonality, Composition, and Antioxidant Capacity of Limonene/δ-3-Carene/(*E*)-Caryophyllene *Schinus terebinthifolia* Essential Oil Chemotype from the Brazilian Amazon: A Chemometric Approach

**DOI:** 10.3390/plants12132497

**Published:** 2023-06-29

**Authors:** Bruna de Araújo Guimarães, Renata Cunha Silva, Eloisa Helena de Aguiar Andrade, William N. Setzer, Joyce Kelly da Silva, Pablo Luis B. Figueiredo

**Affiliations:** 1 Laboratório de Química dos Produtos Naturais, Universidade do Estado do Pará, Belém 66087-670, Brazil; bruna.guimaraes@aluno.uepa.br; 2 Laboratório de Morfofisiologia Aplicada à Saúde, Universidade do Estado do Pará, Belém 66087-670, Brazil; renatacsterapeuta@gmail.com; 3 Laboratório Adolpho Ducke, Coordenação de Botânica, Museu Paraense Emílio Goeldi, Belém 66077-830, Brazil; eloisa@museu-goeldi.br; 4 Aromatic Plant Research Center, 230 N 1200 E, Suite 100, Lehi, UT 84043, USA; joycekellys@ufpa.br; 5 Department of Chemistry, University of Alabama in Huntsville, Huntsville, AL 35899, USA; 6 Programa de Pós-Graduação em Biotecnologia, Universidade Federal do Pará, Belém 66075-900, Brazil; 7 Programa de Pós-Graduação em Ciências Farmacêuticas, Universidade Federal do Pará, Belém 66075-110, Brazil

**Keywords:** Brazilian pepper, chemical variability, Anacardiaceae, volatiles, chemometrics, DPPH, radical-scavenging

## Abstract

*Schinus terebinthifolia* Raddi is widely used in traditional Brazilian medicine to treat respiratory diseases, as an antiseptic, anti-inflammatory, and hemostatic agent. This study aimed to evaluate the influence of climatic parameters on the yield, antioxidative capacity, and chemical composition of the *S. terebinthifolia* leaf essential oil. The specimen was collected monthly from October 2021 to September 2022. Leaf essential oils (EOs) were obtained by hydrodistillation, and their chemical compositions were analyzed by gas chromatography/mass spectrometry (GC/MS). Statistical analyses were performed to verify the climatic influences on the yields, chemical composition, and antioxidative capacity. The DPPH (2,2-diphenyl-1-picrylhydrazyl) radical-scavenging and inhibition of β-carotene/linoleic acid oxidation assays were performed to assess the antioxidant activity. The leaf essential oil yields ranged from 0.1% (July) to 0.7% (May and September), averaging 0.5 ± 0.2%. There was no significant difference in essential oil production during the dry (0.4 ± 0.2%) and rainy (0.6 ± 0.1%) seasons. The main chemical constituents identified in essential oils were limonene (11.42–56.24%), δ-3-carene (8.70–33.16%) and (*E*)-caryophyllene (4.10–24.98%). The limonene annual average was 43.57 ± 12.74% and showed no statistical difference during the dry (40.53 ± 13.38%) and rainy (52.68 ± 3.27%) seasons. Likewise, the annual average of δ-3-carene was 22.55 ± 7.11%, displaying no statistical difference between dry (26.35 ± 7.90%) and rainy (31.14 ± 1.63%) seasons. The annual average of (*E*)-caryophyllene was 11.07 ± 7.15% and this constituent did not show a statistical difference in Tukey’s test (*p* > 0.05) during the dry (12.72 ± 7.56%) and rainy (6.10 ± 1.78%) season. Limonene showed a moderate positive and significant correlation (*p* < 0.05) with precipitation (r = 0.56) and a weak correlation with temperature (r = −0.40), humidity (r = 0.40), and insolation (r = −0.44). All samples inhibited the oxidation in the β-carotene/linoleic acid system (22.78–44.15%) but displayed no activity in the DPPH method.

## 1. Introduction

The Anacardiaceae includes 79 genera with economic potential for providing resins, tannins, and edible fruits such as cashew (*Anacardium occidentale* L.) and mango (*Mangifera indica* L.) [1]. The Anacardiaceae genera are subdivided into five tribes: Anacardieae, Dobineae, Rhoeae, Semecarpeae, and Spondiadeae. Approximately 25% of this family’s genera are known to be toxic, and these are limited to the tribes Anacardieae, Rhoeae, and Semecarpeae. Moreover, phytochemical and biological studies have only been performed on less than 7% of the known Anacardiaceae species [2]. Many taxa are also cultivated as ornamentals, such as the *Schinus* genus [3].

The *Schinus* genus has approximately 37 species, most native to South America [4]. *Schinus* plants are dioecious and female trees are rich sources of potentially active compounds of several secondary metabolites, such as flavonoids, biflavonoids, tannins, catechins, triterpenes, steroids, and essential oils [5]. Many plants in this genus are used in traditional medicine for various diseases, including rheumatism, bronchitis, hypertension, ulcers, abdominal pain, menstrual disorders, gonorrhea, bronchitis, conjunctivitis, dysentery, urinary tract disorders, and eye infections [5].

*Schinus terebinthifolia* Raddi is known as “aroeira-vermelha”, “aroeira-pimenteira”, “Brazilian pepper”, or “pink-pepper” [6]. This tree is native to Paraguay, northeastern Argentina, and Brazil and has been introduced in subtropical areas worldwide [7]. *S. terebinthifolia* leaves contain lanceolate and pointed leaflets, its small flowers are arranged in white or greenish-yellow pedicles, and its fruit is a red drupe with an aroma similar to that of pepper [8]. Moreover, the dried fruit is sold commercially as “pink pepper” [9] and the fruit essential oil (*Schinus molle* L.) is also commercially available [10]. This species is widely used in traditional Brazilian medicine. The leaf is used as an antiseptic, anti-inflammatory, and hemostatic agent [11], and a leaf infusion is used to treat respiratory diseases [12]. In addition, some in-vitro and in-vivo studies have reported biological activities of *S. terebinthifolia* leaf and root extracts such as cytotoxicity against cancer cell lines [13], antioxidant [14], bactericidal, and fungicidal [15,16].

The chemical compositions of essential oils of *Schinus terebinthifolia* have already been described in the literature, presenting germacrene D (33.80%) and (*E*)-caryophyllene (12.25%) as main constituents [12]. However the chemical composition of essential oils is variable, depending on the analyzed plant part, origin, season, and extraction methods, as secondary metabolites can have their biosynthesis affected by natural processes such as plant development, rainfall, seasonality, and temperature of the environment, among other factors that influence the concentration of active constituents [17].

Therefore, considering the chemical and biological potential of *S. terebinthifolia*, this work is aimed to evaluate the influence of seasonality on yield, chemical composition, and antioxidant capacity of a limonene/δ-3-carene/(*E*)-caryophyllene *Schinus terebinthifolia* leaf essential oil chemotype from the Brazilian Amazon.

## 2. Results and Discussion

### 2.1. Essential Oil Yield Seasonal Variation

Two seasons, a dry season and a rainy season, characterize the climate of the Brazilian Amazon, and both are hot and humid. However, the seasons may change from one year to another [18]. The year of this study was atypical, the rainy season lasted only three months (March to May) [19].

**Figure 1:**
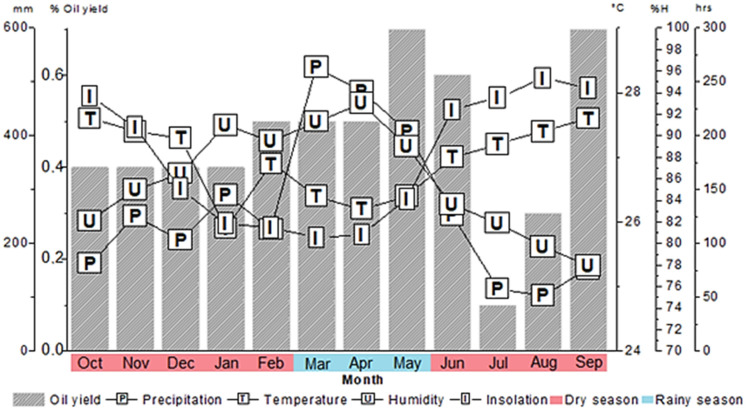
Relationship between climatic parameters and essential oil yield of *Schinus terebinthifolia* during the seasonal study.

The study was carried out from October 2021 to September 2022. During this period, the insolation ranged from a low of 105.4 h in March to a high of 253.4 h in August, the monthly rainfall ranged from 103.9 mm in August to 527.4 mm in March, the average temperatures ranged from 25.9 °C in January to 27.6 °C in October, while the relative humidity ranged from 77.9% (September) to 93.0% (April). According to the rainfall data, the dry season in the region occurred during the months from October to February and June to September, with an average rainfall of 195.8 ± 65.0 mm, and the rainy season from March to May, with an average rainfall of 472.5 ± 60.2 mm (Figure 1).

In this seasonal investigation, the *S. terebinthifolia* leaf essential oil yields (*v*/*w*) ranged from 0.1% (July) to 0.7% (May and September), with an average of 0.5 ± 0.2% during the year of investigation. Statistically (Tukey test), no significant differences in essential oil yield were observed comparing the dry (0.4 ± 0.2%) and rainy (0.6 ± 0.1%) seasons. With respect to the relationship between essential oil yield and climatic parameters, either insignificant or minor correlations were discerned (*p* > 0.05) between the essential oil yield and humidity (r = 0.19), temperature (r = −0.22), and insolation (r = −0.26); precipitation (r = 0.43) displayed weak correlation with essential oil yield (Table 1).

The leaf and fruit essential oil yields of *S. terebinthifolia* sampled in Rio Grande do Sul (southern Brazil) were 0.74 and 0.16% *v*/*w*, respectively [3]. Moreover, the essential oil yield of *S. terebinthifolius* leaves sampled in Minas Gerais State (southeastern Brazil) showed minor changes throughout one year. The essential oil yield ranged from 0.65 to 0.69% in the months of March to September, and 0.45 to 0.55% from October to February, which concurred with the flowering and fruiting stages, respectively [12]. In this study, the average yield was 0.46 ± 0.11% during the flowering stage (October to February, and September) and 0.45 ± 0.2% during the fruiting stage (March to August), showing no significant difference. However, in another specimen collected in El Ghazala, northern Tunisia, the leaves presented a yield of 1.06% (*w*/*w* on dry weight) [20]. On the other hand, Santana et al. [21] collected the fresh leaves of *S. terebinthifolia* in Diadema City, São Paulo (southeastern Brazil), and obtained 571 mg of crude essential oil (yield of 0.17%).

### 2.2. Seasonal Effects in Essential Oil Chemical Composition

The 52 volatile components in the essential oils of the leaves of *S. terebinthifolia*, identified by GC/MS and quantified by GC-FID, are presented in Table 2, which are listed in order of their elution from the GC. The identified components comprise 98.06–99.93% of the total essential oil compositions in this seasonal investigation.

The predominant classes in the leaf samples were the monoterpene hydrocarbons (21.85–93.35%) and sesquiterpene hydrocarbons (6.46–67.82%), followed by oxygenated sesquiterpenes (0.01–5.75%) and oxygenated monoterpene (0.09–1.06%). The main chemical constituents (>5%) identified in essential oils from this seasonal study were the monoterpene hydrocarbons limonene, which ranged from 11.42% (July) to 56.24% (May) and δ-3-carene, which ranged from 8.70% (July) to 33.16% (September). The sesquiterpene hydrocarbons (*E*)-caryophyllene ranged from 4.10% (May) to 24.98% (July); α-copaene ranged from 1.11% (September) to 8.32% (July); and β-selinene ranged from 0.27% (May) to 5.13% (July). Limonene was the major constituent in *S. terebinthifolia* leaf essential oil in this study, except in July, where the major component was (*E*)-caryophyllene (24.98%).

The limonene annual average was 43.57 ± 12.74%, displaying no statistical difference (Tukey’s test, *p* > 0.05) during the dry (40.53 ± 13.38%) and rainy (52.68 ± 3.27%) seasons. Likewise, the annual average of δ-3-carene was 22.55 ± 7.11%; and nonstatistical difference in Tukey’s test (*p* > 0.05) during the dry (26.35 ± 7.90%) and rainy (31.14 ± 1.63%) seasons. Thus, the annual average of (*E*)-caryophyllene was 11.07 ± 7.15% and this constituent did not show a statistical difference in Tukey’s test (*p* > 0.05) during the dry (12.72 ± 7.56%) and rainy (6.10 ± 1.78%) season.

Based on Pearson’s correlation coefficient analysis (Table 1), limonene showed a moderate positive and significant correlation (*p* < 0.05) with precipitation (r = 0.56) and a weak correlation with temperature (r = −0.40), humidity (r = 0.40) and insolation (r = −0.44). The other major constituent, δ-3-carene, showed a weak correlation with the climatic parameters of precipitation (r = 0.46), temperature (r = −0.33), humidity (r = 0.32), and insolation (r = −0.35). (*E*)-Caryophyllene showed a moderate negative correlation with precipitation (r = −0.54), weak negative correlation with humidity (r = −0.37) and weak positive correlations with insolation (r = 0.39) and temperature (r = 0.43).

The chemical composition of *S. terebinthifolia* leaf essential oil sampled in Porto Velho, Rondônia presented the sesquiterpenes germacrene D (25.0%), (*E*)-caryophyllene (17.5%), and δ-elemene (10.5%) as main constituents [22]. A study made on ripe fruits of *S. terebinthifolia* collected in Vitória, Espírito Santo presented monoterpenes (85.1%) as the predominant class, the most abundant were δ-3-carene (30.4%), limonene (17.4%), α-phellandrene (12.6%), and α-pinene (12.6%) [23].

(*E*)-caryophyllene essential oil concentrations have displayed correlations with environmental parameters. An (*E*)-caryophyllene-rich *Eugenia patrisii* Vahl specimen (Myrtaceae) monitored for one year showed a correlation with humidity (r = −0.49) and insolation (r = 0.48) and temperature (r = 0.65) [24]. Moreover, (*E*)-caryophyllene acts as the core of plant signaling networks, inducing resistance against microbial pathogens in neighboring plants via jasmonic acid (JA)-signaling. Thus, (*E*)-caryophyllene functions as an allelochemical component in complex plant signaling networks [25]. 

**Table 2:** Chemical composition of essential oils from leaves of *Schinus terebinthifolia* during the seasonal study.

RI_(C)_	RI_(L)_	Month	Oct.	Nov.	Dec.	Jan.	Feb.	Mar.	Apr.	May	Jun.	Jul.	Aug.	Sep.
		Essential Oil Yield	0.4	0.4	0.4	0.4	0.5	0.5	0.5	0.7	0.6	0.1	0.3	0.7
		Constituents ^c^	(%) *											
929	932 ^a^	α-Pinene	0.78	0.91	0.39	0.55	1.55	0.79	1.59	1.61	1.64	0.60	0.44	1.41
946	946 ^a^	Camphene							0.09		0.11			
987	988 ^a^	Myrcene	1.44	2.67	0.56	1.78	2.47	2.03	2.07	2.79	2.58	0.42	0.84	2.63
**1009**	**1008 ^a^**	**δ-3-Carene**	**26.40**	**29.05**	**19.45**	**30.66**	**32.84**	**29.32**	**32.47**	**31.64**	**31.32**	**8.70**	**25.60**	**33.16**
1018	1014 ^a^	α-Terpinene										0.18		
1023	1020 ^a^	*p*-Cymene				0.18	0.03		0.25	0.28	0.18		0.11	0.02
**1029**	**1024 ^a^**	**Limonene**	**44.05**	**43.28**	**29.56**	**49.27**	**52.80**	**51.99**	**49.81**	**56.24**	**45.20**	**11.42**	**35.58**	**53.64**
1043	1044 ^a^	(*E*)-β-Ocimene		1.05	0.14	0.66	0.09	0.20	0.23	0.12	1.06		0.19	0.25
1055	1054 ^a^	γ-Terpinene		0.27		0.20	0.27	0.30	0.08	0.14	0.22	0.14		0.32
1079	1085 ^a^	*p*-Mentha-2,4(8)-diene						0.05		0.05	0.06			0.06
1084	1086 ^a^	Terpinolene	0.49	0.67	0.27	0.54	0.59	0.71	0.41	0.43	0.66	0.39	0.48	0.82
1089	1089 ^a^	*p*-Cymenene	0.16	0.13	0.15	0.14	0.12		0.10	0.05	0.09		0.16	
1098	1095 ^a^	Linalool		0.30	0.55	0.45		0.13	0.12	0.08	0.10			0.17
1112	1113 ^b^	4,8-Dimethyl-(*E*)-nona-1,3,7-triene	0.24	0.32	0.56	0.42	0.03	0.14	0.20		0.18		0.19	0.08
1193	1186 ^a^	α-Terpineol		0.09		0.14	0.03	0.03	0.06		0.06		0.13	
1197	1200 ^a^	*trans*-Dihydrocarvone				0.15			0.14		0.08	0.21	0.44	
1217	1215 ^a^	*trans*-Carveol	0.26	0.15	0.27	0.17	0.06	0.06	0.12	0.03	0.18		0.30	0.09
1229	1226 ^a^	*neoiso*-Dihydrocarveol											0.19	
1242	1239 ^a^	Carvone						0.01	0.04					
1374	1374 ^a^	α-Copaene	3.59	2.76	6.27	2.43	1.50	2.14	2.17	1.19	2.33	8.32	4.70	1.11
1388	1389 ^a^	β-Elemene		0.05				0.01			0.02		0.07	
**1420**	**1417 ^a^**	**(*E*)-Caryophyllene**	**13.49**	**9.79**	**23.16**	**7.74**	**5.13**	**7.48**	**6.73**	**4.10**	**8.54**	**24.98**	**17.20**	**4.44**
1433	1437 ^a^	α-Guaiene	0.05									0.55		
1436	1439 ^a^	Aromadendrene	0.35	0.33	0.89	0.19	0.12	0.19	0.17	0.06	0.23	1.84	0.53	0.07
1447	1449 ^a^	α-Himachalene	0.11								0.05	0.30	0.09	
1454	1452 ^a^	α-Humulene	1.14	0.92	2.20	0.61	0.37	0.60	0.53	0.25	0.77	3.25	1.72	0.30
1458	1458 ^a^	*allo*-Aromadendrene		0.05	0.14			0.02			0.04	0.39	0.05	
1473	1478 ^a^	γ-Muurolene	1.03	0.56	1.34	0.38	0.18	0.35	0.27	0.11	0.59	3.71	1.02	0.12
1478	1483 ^a^	α-Amorphene	0.08	0.05	0.14	0.03		0.03			0.06	0.55	0.10	
1487	1489 ^a^	β-Selinene	1.70	1.39	3.37	0.90	0.53	0.86	0.70	0.27	1.02	5.13	2.08	0.3
1494	1498 ^a^	α-Selinene	1.28	1.02	2.38	0.69	0.40	0.64	0.51	0.22	0.75	6.20	1.48	0.22
1496	1500 ^a^	α-Muurolene	0.38	0.28	0.57	0.18	0.10	0.16	0.15	0.05	0.21	2.23	0.54	0.06
1499	1509 ^a^	α-Bulnesene	0.20											
1500	1495 ^a^	*cis*-Cadina-1,4-diene						0.01						
1503	1505 ^a^	(*E,E*)-α-Farnesene		0.05	0.10			0.02					0.05	0.01
1511	1513 ^a^	γ-Cadinene	0.34	0.29	0.68	0.15	0.08	0.15	0.13	0.04	0.19	1.69	0.48	0.05
1516	1513 ^a^	δ-Cadinene	1.28	1.05	2.5	0.66		0.64	0.49	0.17	0.80	6.24	1.92	0.27
1516	1511 ^a^	δ-Amorphene					0.38							
1519	1521 ^a^	*trans*-Calamenene							0.03				0.26	
1521	1528 ^a^	Zonarene			0.18			0.04						
1530	1533 ^a^	*trans*-Cadina-1,4-diene	0.11	0.12	0.23	0.05	0.03	0.05			0.08	0.48	0.17	
1534	1537 ^a^	α-Cadinene	0.11	0.08	0.19			0.03			0.04	0.53	0.13	
1538	1545 ^a^	Selina-4(15),7(11)-diene										0.31		
1539	1544 ^a^	α-Calacorene	0.11	0.06	0.18			0.02			0.03	1.12	0.17	
1559	1559 ^a^	Germacrene B			0.09									
1559	1561 ^a^	(*E*)-Nerolidol		0.04									0.04	
1572	1570 ^a^	Caryophyllene alcohol ^d^	0.42	0.34	0.72	0.09		0.05			0.09	0.53		0.02
1579	1582 ^a^	Caryophyllene oxide	0.11	0.26	0.37	0.07	0.01	0.05	0.09	0.01	0.05	2.32	0.25	
1629	1630 ^a^	γ-Eudesmol		0.07	0.18	0.04		0.02				1.46	0.18	
1652	1649 ^a^	β-Eudesmol										0.30		
1653	1652 ^a^	α-Cadinol		0.08	0.22	0.03						1.14	0.18	
1656	1658 ^a^	Selin-11-en-4α-ol		0.03	0.08									
Monoterpene hydrocarbons			73.32	78.03	50.52	83.98	90.76	85.39	87.10	93.35	83.12	21.85	63.4	92.31
Oxygenated monoterpenes			0.26	0.54	0.82	0.91	0.09	0.23	0.48	0.11	0.42	0.21	1.06	0.26
Sesquiterpene hydrocarbons			25.35	18.85	44.61	14.01	8.82	13.44	11.88	6.46	15.75	67.82	32.76	6.95
Oxygenated sesquiterpenes			0.53	0.82	1.57	0.23	0.01	0.12	0.09	0.01	0.14	5.75	0.65	0.02
Others			0.24	0.32	0.56	0.42	0.03	0.14	0.20	0.00	0.18	0.00	0.19	0.08
Total identified (%)			99.70	98.56	98.08	99.55	99.71	99.32	99.75	99.93	99.61	95.63	98.06	99.62

RI_(C)_: Calculated Retention Index; RI_(L)_: Literature Retention Index; **^a^**: Adams, 2007 [26]; **^b^**: FFNSC [27]; **^c^**: Mass spectra similarity indices higher than 90%; ^d^: 4,4,8-trimethyltricyclo[6.3.1.0^2,5^]dodecan-1-ol. Main constituents in bold, * n = 2 (standard deviation was less than 2.0).

**Figure 2:**
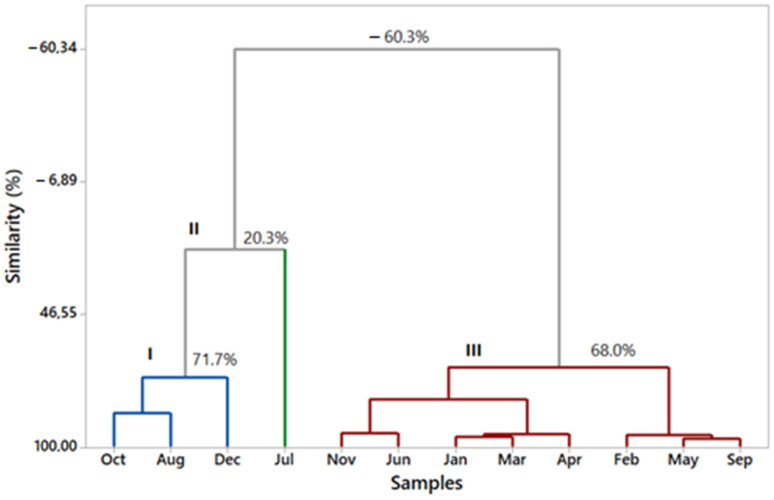
Dendrogram demonstrating the similarities between the essential oil compositions of *Schinus terebinthifolia* during the seasonal investigation.

A hierarchical clustering analysis (HCA) using the essential oil components (>2.0%) was carried out (Figure 2). The HCA showed the compositions of the essential oils analyzed to be grouped into three different chemical profiles and exhibited no similarity between them. Profile I, which displayed 71.7% similarity between the samples, comprises the leaf essential oils collected in October, August, and December, whose main constituents were limonene (36.40 ± 7.28%), δ-3-carene (23.82 ± 3.80%), (*E*)-caryophyllene (17.95 ± 4.88%), and α-copaene (4.85 ± 1.35%). Profile II includes the essential oil sample for July, which presented (*E*)-caryophyllene (24.98%), limonene (11.42%), δ-3-carene (8.70%), α-copaene (8.32%), δ-cadinene (6.24%), α-selinene (6.20%), and β-selinene (5.13%). Profile III, which grouped November, June, January, March, April, February, May, and September, whose main constituents were limonene (50.3 ± 4.3%), δ-3-carene (31.31 ± 1.54%), and (*E*)-caryophyllene (6.74 ± 2.03%) displayed 68.00% similarity between the samples.

**Figure 3:**
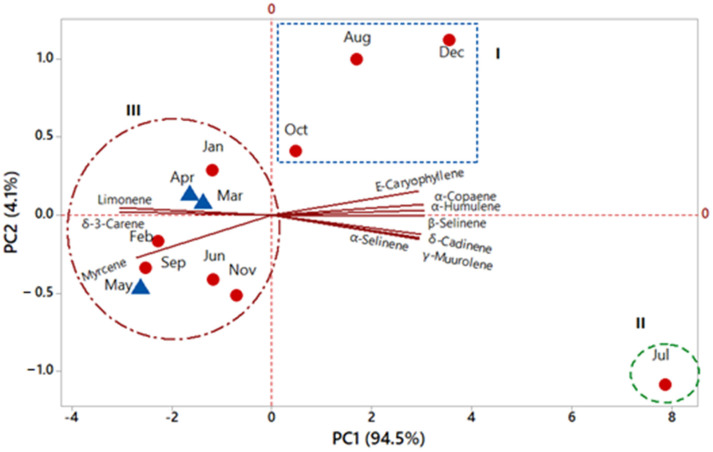
Principal components analysis of the essential oils of *Schinus terebinthifolia* in the seasonal study.

In the principal component analysis (PCA, Figure 3), the principal components PC1 and PC2 accounted for 98.6% of the total data variability. PC1 described 94.5% and displayed negative correlations with the variables myrcene (r = −0.29), limonene (r = −0.32), and δ-3-carene (r = −0.32) and positive correlations with α-copaene (r = 0.32), (*E*)-caryophyllene (r = 0.31), α-humulene (r = 0.32), γ-muurolene (r = 0.32), β-selinene (r = 0.32), α-selinene (r = 0.31), and δ-cadinene (r = 0.32). PC2 explained 4.1% and displayed negative correlation with the variables myrcene (r = −0.67), γ-muurolene (r = −0.36), β-selinene (r = −0.02), α-selinene (r = −0.38), and δ-cadinene (r = −0.30); positive correlations with limonene (r = 0.06), δ-3-carene (r = 0.11), α-copaene (r = 0.16), (*E*)-caryophyllene (r = 0.37), and α-humulene (r = 0.07). Both HCA and PCA analyses indicated no separation between the essential oil samples of *Schinus terebinthifolia* during the dry and rainy periods.

**Figure 4:**
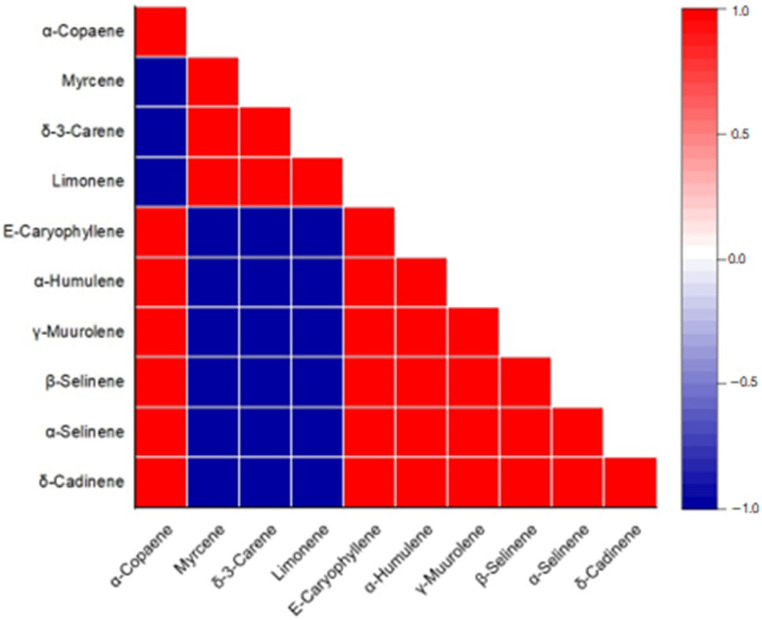
Heat correlation map between the main constituents.

Based on Pearson’s correlation coefficient analysis (Table A1 and Figure 4), all the compounds (≥5%) showed a significant correlation (*p* < 0.05) between themselves. Limonene presented strong and negative correlation with α-copaene (r = −0.98), (*E*)-caryophyllene (r = −0.95), and β-selinene (r = −0.99) and strong positive correlation with δ-3-carene (r = 0.96). δ-3-carene presented strong negative correlation with α-copaene (r = −0.97), (*E*)-caryophyllene (r = −0.93), and β-selinene (r = −0.99). (*E*)-caryophyllene showed a strong and positive correlation with α-copaene (r = 0.98) and β-selinene (r = 0.96).

**Figure 5:**
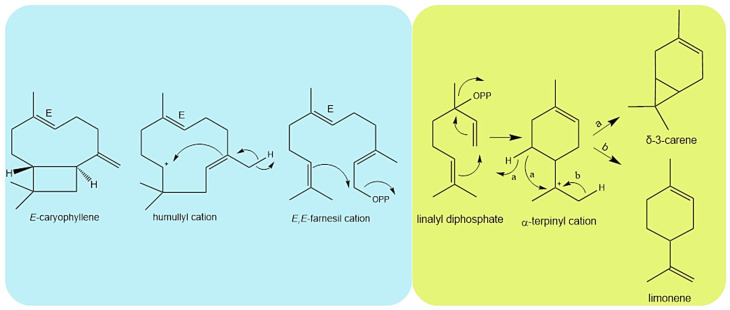
Biosynthetic pathway of (*E*)-caryophyllene, δ-3-carene and limonene.

Limonene and (*E*)-caryophyllene arise from different biosynthetic cations (Figure 5), which explains why in July, for example, (*E*)-caryophyllene presented the major content while limonene presented the lower content, the opposite happened in May. The same thing happened to δ-3-carene and (*E*)-caryophyllene; in July, there was a lower concentration of δ-3-carene and a higher concentration of (*E*)-caryophyllene; February presented the higher content of δ-3-carene and a low content of (*E*)-caryophyllene. On the same line of thought, limonene and δ-3-carene arise from to same biosynthetic precursor cation, it is noticeable that from October to November the content of δ-3-carene increases and limonene decreases. Moreover, α-copaene and β-selinene belong to the biosynthetic precursor of germacryl cation.

### 2.3. Antioxidant Capacity

The antioxidant capacities of the essential oil samples were evaluated using two different assays. All samples inhibited the oxidation in the β-carotene/linoleic acid system (22.78–44.15%) (Table 3), while the DPPH radical scavenging assay showed no inhibition.

The greater inhibition was observed in the essential oil sampled in August (44.15 ± 3.0%) and June (37.62 ± 2.74%), with only half of the Trolox standard inhibition (82.93 ± 1.8%). The months of October to May showed no statistical difference in the Tukey test (*p* > 0.05), with inhibition between 22.77 to 30.65%. Hassimotto et al. defined that a percentage of oxidation inhibition between 40 and 70% characterizes an intermediate antioxidant capacity [28]. Furthermore, there was a weak correlation between limonene amounts and a negligible correlation between δ-3-carene and (*E*)-caryophyllene and antioxidant capacity.

In the β-carotene/linoleic acid assay, β-carotene rapidly changes color in the absence of antioxidants. This is due to the coupled oxidation of β-carotene and linoleic acid, which generates free radicals. Formed by abstracting a hydrogen atom from its diallylmethylene group, the linoleic acid radical attacks a highly unsaturated β-carotene molecule. As a result, β-carotene is oxidized and partially degraded, subsequently losing its chromophore and its characteristic orange color [29]. However, the DPPH assay is based on radical scavenging; when a compound that can donate a hydrogen atom is mixed with a solution of DPPH, the DPPH radical is reduced with concomitant loss of the violet color, then, the free radical formed can undergo additional reactions to create a stable product. While DPPH can either accept a hydrogen atom or an electron to form a stable, diamagnetic molecule, and oxidation of DPPH is difficult and irreversible [30].

Monoterpene-rich extracts have demonstrated antioxidant activity against DPPH, although, when only limonene was tested, it was less reactive [31]. Different concentrations of δ-3-carene were tested in the DPPH assay, the higher inhibition (4.8 ± 0.4%) occurred at 4 µg/mL, showing low activity [32]. (*E*)-Caryophyllene showed a weak antioxidant efficacy in the DPPH method (IC_50_ 132.0 ± 9.9 µg/mL); however it was effective in antioxidant chain breaking in lipid peroxidation in vitro and had greater radical-scavenging behavior with reactive oxygen species than with relatively stable organic radicals [33]. Therefore, the low antioxidant capacity of *Schinus terebinthifolia* essential oil can be rationalized by the low capacity of the major components. 

## 3. Materials and Methods

### 3.1. Plant Material and Climatic Data

The leaves of *Schinus terebinthifolia* were collected from a single specimen in Belém city, Pará state, Brazil (coordinates: 1°27′13.4″ S/48°29′34.1″ W). For the seasonal study, the mature leaves (150 g) were sampled on day 30 of each month, at 3 pm, from October 2021 to September 2022. Plant identification was performed by comparison with an authentic specimen of *Schinus terebinthifolia* Raddi, and a plant sample was deposited with the Herbarium “João Murça Pires”, at Museu Paraense Emílio Goeldi, Belém city, State of Pará, Brazil (MG-245400). The specimen was collected in agreement with Brazilian laws concerning biodiversity protection (A075D38).

During the collection period, the climatic parameters (insolation, relative air humidity, and rainfall precipitation) of the collection site were obtained each month from the website of the Instituto Nacional de (INMET, http://www.inmet.gov.br/portal/, accessed on the 24 October 2022, of the Brazilian Government (INMET, 2022) [34]. Meteorological data were recorded through the automatic station A-201, located in Belém city, Pará state, Brazil, equipped with a Vaisala system, model MAWS 301 (Vaisala Corporation, Helsinki, Finland) [19].

### 3.2. Extraction and Essential Oil Composition

The leaves of *S. terebinthifolia* were air-dried and 150-g samples were pulverized and hydrodistillation using a Clevenger-type apparatus for 3 h. The hydrodistillation was repeated twice for each sample. The essential oils were dried over anhydrous sodium sulfate, and the masses of dry plant material were used to calculate the essential oil yields. The moisture content of the plant samples was determined using an infrared moisture balance for water loss measurement. Analysis of essential oil yield was conducted in duplicate. The essential oil was dissolved in *n*-hexane (1500 µg/mL, 3:500, *v*/*v*) and analyzed by gas chromatography–flame ionization detector (GC-FID, Shimadzu Corporation, Tokyo, Japan) and gas chromatography-mass spectrometry (GC/MS, Shimadzu Corporation, Tokyo, Japan) simultaneously using the two systems. The essential oil analyses were performed in a GCMS-QP2010 system (Shimadzu Corporation, Tokyo, Japan), equipped with an AOC-20i auto-injector and the GCMSSolution software that included both the Adams and FFNSC-2 libraries [26,27]. The GC column used was an Rxi-5ms (30 m; 0.25 mm; 0.25 µm film thickness) silica capillary column (Restek Corporation, Bellefonte, PA, USA). The following operating conditions for the analysis were injector temperature = 250 °C; oven temperature programming was 60–250 °C at a rate of 3 °C/min); helium was used as the carrier gas, which was set to a linear velocity of 36.5 cm/s (1.0 mL/min); 1.0 µL of essential oil solution (6 µg of essential oil injected) was injected using a splitless mode of injection; ionization by electronic impact at 70 eV; the ionization source temperature was 220 °C and the transfer line temperature was 250 °C. The mass spectra were obtained using a scan range of 40–450 m/z and a scan rate of 2.0 scans/sec. The retention indices were calculated for all volatile components based on a homologous series of C8-C40 *n*-alkanes (Sigma-Aldrich, Milwaukee, WI, USA), according to the linear equation of van Den Dool and Kratz [35]. Each Individual component was identified by comparing its retention index and mass spectral and fragmentation pattern with those found in the GCMS-Solution system libraries. The quantitative data regarding the volatile constituents were obtained using a GC 2010 Series instrument with a flame ionization detector, operated under similar conditions to the GC-MS system, detector temperature of 250 °C. The percent compositions of individual components were calculated by peak-area normalization without a response factor using the flame ionization detector (GC-FID). The GC-FID and GC/MS analyses were carried out in duplicate.

### 3.3. Antioxidant Evaluation

#### 3.3.1. β-Carotene/Linoleic Acid Assay

The stock solution of β-carotene/linoleic acid mixture was prepared by dissolving 0.2 mg of β-carotene in 1 mL of HPLC grade chloroform, followed by the addition of 20 μL of linoleic acid and 200 mg of Tween 20. The chloroform was then completely evaporated under reduced pressure. Then, 50 mL of oxygenated water was added with vigorous agitation. Aliquots (2500 μL) of the β-carotene/linoleic acid reaction mixture were distributed into test tubes and 200-μL portions of the essential oil samples (1.0 mg/mL in ethanol) were added. The emulsion systems were incubated at 50 °C. The same procedure was carried out using Trolox and a blank of ethanol as the control. The absorbances of the solutions were recorded at 470 nm and monitored at intervals of 15 min, for 120 min. The antioxidant activity (AA%) was calculated as the percent inhibition relative to the control using $AA\%=[1-({Abs}_{sample}^{0}-{Abs}_{sample}^{120})/({Abs}_{control}^{0}-{Abs}_{control}^{120})]\times 100$. All tests were carried out in triplicate [36].

#### 3.3.2. 2,2-Diphenyl-1-Picrylhydrazyl (DPPH) Assay

The stable dark-violet 2,2-diphenyl-1-picrylhydrazyl (DPPH) free radical has a maximum absorption at 517 nm, which is reduced in the presence of antioxidants. A DPPH stock solution (0.5 mM) was prepared in ethanol. The stock solution was diluted to approximately 60 μM and showed an initial absorbance of 0.62 ± 0.02 at 517 nm at room temperature. Each essential oil sample from the seasonal study (50 μL, 10 mg/mL) was mixed with Tween 20 solution (0.5%, 50 μL, *w*/*w*) and then added to the DPPH (0.5 mM, 1900 μL) in ethanol. For each sample, an ethanol control blank was also measured. The absorbance was measured (Ultrospec™ 7000, Biochrom US, Holliston, MA, USA) at the start of the reaction (time zero), every 5 min during the first 30 min, and then at 30 min intervals until constant absorbance values were observed (plateau of reaction, 2 h). A Trolox (6-hydroxy-2,5,7,8-tetramethylchroman-2-carboxylic acid) (Sigma-Aldrich, St. Louis, MO, USA) standard curve was prepared using concentrations of 30, 60, 150, 200, and 250 μg/mL. The DPPH free-radical inhibitions were expressed as milligrams of Trolox (mg TE/g) equivalents per gram of the sample [37,38].

### 3.4. Statistical Analysis

Statistical significance was evaluated using the Tukey test (*p* < 0.05). Pearson correlation analyses were carried out to determine the relationship between the major essential oil components (δ-3-carene, limonene, α-copaene, (*E*)-caryophyllene, α-humulene, γ-muurolene, β-selinene, α-selinene, and δ-cadinene) and the climatic parameters analyzed (insolation, relative air humidity, temperature, and rainfall precipitation), using the software GraphPad Prism, version 5.0. The principal component analysis (PCA) was utilized to verify the interrelation in the essential oil components (>2.0%) using the Minitab^®^ software (free 390 Version, Minitab Inc., State College, PA, USA). The hierarchical cluster analysis (HCA) was carried out using the Euclidean distance and Ward linkage to verify the similarity of the essential oil samples based on the distribution of the constituents selected in the previous PCA analysis [38].

## 4. Conclusions

The *Schinus terebinthifolia* essential oil yield is not correlated with climatic parameters, showing no statistical difference between the rainy and dry seasons. Limonene and δ-3-carene were the main compounds throughout the study period, except in July, when the main constituent was (*E*)-caryophyllene, with quantitative variations in their concentration, which characterize a chemotype yet not described in the literature.

Moreover, all the samples inhibited the oxidation in the β-carotene/linoleic acid system and there was a weak or negligible correlation between limonene and δ-3-carene amounts and antioxidant capacity.

Thus, the variation in the content of the main constituents was not explained/correlated to the climatic parameters. Since there were quantitative and qualitative variations in the chemical composition of *S. terebinthifolia* essential oil, future studies focusing on seasonality, comparison between different plant tissues, antifungal, antibacterial, and other biological activities would be informative. A prior understanding of the phytochemical variations of the plant is necessary to appreciate the medicinal utility of *S. terebinthifolia*.

## Figures and Tables

**Table 1:** Correlation between *Schinus terebinthifolia* essential oil yield, major components, and climatic parameters.

Parameter	Essential Oil Yield	δ-3-Carene	Limonene	(*E*)-Caryophyllene
Temperature	−0.22	−0.33	−0.40	0.43
Humidity	0.19	0.32	0.40	−0.37
Insolation	−0.26	−0.35	−0.44	0.39
Precipitation	0.43	0.46	0.56 *	−0.54

* Significant at correlation (*p* < 0.05).

**Table 3:** Antioxidant capacity of the monthly essential oils of *Schinus terebinthifolia* in β-carotene/linoleic acid system.

Sample	Inhibition (%)
October	26.16 ± 3.69 ^a,b^
November	24.58 ± 3.56 ^a^
December	28.29 ± 1.57 ^a,b^
January	26.00 ± 2.90 ^a^
February	24.90 ± 2.04 ^a^
March	22.77 ± 4.37 ^a^
April	30.65 ± 1.35 ^a,b,c^
May	24.04 ± 2.28 ^a^
June	37.62 ± 2.74 ^c,d^
August	44.15 ±3.05 ^d^
September	34.31 ± 2.29 ^b,c^
Trolox	82.93 ± 1.82 ^e^

Mean ± Standard deviation. Values with the same letters in the column do not differ statistically in the Tukey test (*p* > 0.05).

## Data Availability

Data are available from the corresponding author (P.L.B.F.) upon reasonable request.

## Abbreviations List

GC/MS	Gas chromatography-mass spectrometry
GC-FID	Gas chromatography–flame ionization detector
HCA	Hierarchical cluster analysis
PCA	Principal component analysis
R	Pearson’s correlation coefficient
RI_(C)_	Calculated Retention Index
RI_(L)_	Literature Retention Index

## Appendix A

**Table A1:** Correlation between chemical components (≥2%) of *Schinus terebinthifolia* essential oil.

	Myrcene	δ-3-Carene	Limonene	α-Copaene	(*E*)-Caryophyllene	α-Humulene	γ-Muurolene	β-Selinene	α-Selinene
δ-3-carene	0.84								
limonene	0.84	0.96							
α-copaene	−0.91	−0.97	−0.98						
(*E*)-caryophyllene	−0.93	−0.93	−0.95	0.98					
α-humulene	−0.89	−0.98	−0.99	0.99	0.97				
γ-muurolene	−0.77	−0.97	−0.96	0.93	0.87	0.95			
β-selinene	−0.86	−0.99	−0.99	0.99	0.96	0.99	0.96		
α-selinene	−0.76	−0.97	−0.96	0.93	0.86	0.94	0.99	0.96	
δ-cadinene	−0.79	−0.98	−0.97	0.95	0.89	0.96	0.99	0.97	0.99

All correlation were significative (*p* < 0.05).
